# Retraction: Fecal microbiota transplantation for treatment of recurrent *C*. *difficile* infection: An updated randomized controlled trial meta-analysis

**DOI:** 10.1371/journal.pone.0316040

**Published:** 2024-12-13

**Authors:** The PLOS ONE Editors

After this article [1] was published, concerns were raised about the methods and findings of this meta-analysis.

Specifically:

1. The title of this study [1] does not accurately describe the research conducted in this meta-analysis. Instead, the title should have clarified that the study compares fresh fecal microbiota transplant treatments against frozen fecal microbiota transplant treatments and other interventions for the treatment of recurrent *Clostridium difficile* infection (RCDI), as opposed to suggesting that this meta-analysis compares general fecal microbiota transplants (which also include frozen samples) against other interventions.
2. The introduction of [1] states “In recent years, fecal microbiota transplantation (FMT) has been more effective than traditional treatment for RCDI patients, especially fresh FMT.”. The authors did not provide any references supporting the statement that fresh FMT is more effective than frozen FMT. In contrast, several previous studies demonstrated that FMT is equally effective in treating RCDI irrespective of fresh or frozen fecal specimens used [2–4].
3. This study [1] did not include unpublished trials and abstracts, and instead criticized [5] for the inclusion of non-peer-reviewed material such as meeting summaries and abstracts. Although it is considered acceptable to only include peer-reviewed material in meta-analyses, the Cochrane Handbook for Systematic Reviews of Interventions states that “Finding out about unpublished studies, and including their results in a systematic review when eligible and appropriate, is important for minimizing bias.” [6]. As such, criticism that a meta-analysis is flawed because it includes material that was not peer-reviewed is inappropriate.
4. The meta-analysis’ protocol for this study [1] was not registered in PROSPERO, and it is unclear whether the primary outcome of the study or the subgroups were defined a priori.
5. The article [1] uses the terms “relapse”, “remission”, and “recurrence” interchangeably, even though these terms each have distinct meanings in medical contexts.
6. The serious adverse event assessment reported in [1] is insufficient.

The article incorrectly states that the overall quality of evidence in [1] was conducted based on the GRADE approach. Risk of bias in included studies was assessed using the Cochrane Collaboration’s tool for assessing risk of bias in randomized trials [7]. However, the article does not include any analyses based on the GRADE approach, and it is not possible to draw any conclusions about the certainty in the body of evidence based on the results presented.The corresponding author responded to the above concerns. They disagree with point 1, 6 and 7, stating the article’s title is both fitting and descriptive of the research presented and that the study adhered to the methodological standards for meta-analyses, including the assessment of adverse events. In addition, although the authors acknowledge that the article does not report a GRADE analysis, they state that the reported research was conducted in accordance with GRADE principles.

The corresponding author acknowledges the issues described in point 2, 3 4, and 5. They agree that the effectiveness of FMT, particularly fresh FMT, over traditional treatments may have been overstated, and that the criticism on the inclusion of non-peer-reviewed materials in other studies is not in line with recommended guidance presented in the Cochrane Handbook for Systematic Reviews of Interventions [6]. In addition, the corresponding author agreed that the terms "relapse," "remission," and "recurrence" were not clearly defined or used consistently throughout the article.

After editorial assessment of the above, the *PLOS ONE* Editors found that the corresponding author’s responses did not fully resolve the concerns and that the article does not meet *PLOS ONE’s* publication criteria (#3, #4 and #5). We regret that the issues with the article were not identified and addressed prior to its publication.

In light of the above concerns, the *PLOS ONE* Editors retract this article.

WH did not agree with the retraction and stands by the article’s findings. TL, WL, CZ and FG either did not respond directly or could not be reached.
